# Discrepancies between Judgment and Choice of Action in Moral Dilemmas

**DOI:** 10.3389/fpsyg.2013.00250

**Published:** 2013-05-16

**Authors:** Sébastien Tassy, Olivier Oullier, Julien Mancini, Bruno Wicker

**Affiliations:** ^1^Institut de Neurosciences de la Timone, CNRS UMR 7289, Aix-Marseille UniversitéMarseille, France; ^2^Assistance Publique – Department of Psychiatry, Hôpitaux de Marseille, Sainte Marguerite University HospitalMarseille, France; ^3^Cognitive Psychology Lab, UMR CNRS 7920, Research Federation 3C (FR 3512), Aix-Marseille UniversitéMarseille, France; ^4^Teaching and Research Laboratory of Medical Information (LERTIM), School of Medicine, Aix Marseille UniversityMarseille, France; ^5^Assistance Publique – Hôpitaux de Marseille, La Timone University Hospital, Public Health and Medical Information Department (SSPIM)Marseille, France; ^6^Integrative Neurosciences Laboratory, Physics Department, University of Buenos AiresBuenos Aires, Argentina

**Keywords:** moral cognition, dilemma, utilitarianism, affective proximity, decision making

## Abstract

Everyone has experienced the potential discrepancy between what one judges as morally acceptable and what one actually does when a choice between alternative behaviors is to be made. The present study explores empirically whether judgment and choice of action differ when people make decisions on dilemmas involving moral issues. Two hundred and forty participants evaluated 24 moral and non-moral dilemmas either by judging (“*Is it acceptable to*…”) or reporting the choice of action they would make (“*Would you do*…”). We also investigated the influence of varying the number of people benefiting from the decision and the closeness of relationship of the decision maker with the potential victim on these two types of decision. Variations in the number of beneficiaries from the decision did not influence judgment nor choice of action. By contrast, closeness of relationship with the victim had a greater influence on the choice of action than on judgment. This differentiation between evaluative judgments and choices of action argues in favor of each of them being supported by (at least partially) different psychological processes.

## Introduction

Many important individual or social moral decisions require the evaluation of dilemmas leading to outcomes of variable values and consequences. In the domain of moral cognition, prototypical experimental settings often use hypothetical contexts in which individuals have to make life and death decisions in a range of circumstances designed to pit various principles against one another (Greene et al., 2001; Valdesolo and DeSteno, 2006). Moral philosophers, evolutionary biologists, and psychologists have identified several critical factors that may influence moral decision making, such as the distinction between action and omission or the distinction between harm as a means and harm as a side-effect (Cushman et al., 2006; Schaich Borg et al., 2006; Bartels, 2008). Other studies have confirmed that many dimensions incorporated into the hypothetical dilemmas systematically influenced participants’ responses (Christensen and Gomila, 2012). For instance, people are more inclined to approve of options that spare the greater number of individuals, options that spare kin or friends, and options that spare humans vs. non-humans (Petrinovich et al., 1993; O’Neill and Petrinovich, 1998; Nichols and Mallon, 2006). In most studies, participants were required to evaluate moral dilemmas by answering various questions, ranging from “*Is it acceptable to*…” to “*Would you do*…*in order to*….” Interestingly, these different questions may target different psychological processes (Monin et al., 2007) that potentially rely on distinct neural underpinnings (Schaich Borg et al., 2006). Choice differs from judgment because it implies projecting oneself in a direct interaction using an egocentric frame *of* reference with potential self-relevant consequences as emphasized by Sood and Forehand (2005). By contrast, judging relies on an evaluation of the situation from a more allocentric perspective (Frith and de Vignemont, 2005). It is important to note that both types of decision making may rely on normatives aspects and address the commonsense notion that if something is right, one should accept to/do it (Manstead, 2000). Therefore the distinction between judgment and personal action taking may appear quite fundamental (FeldmanHall et al., 2012). Recently, several experiments have provided evidence that this difference between ego- and allocentric perspectives should be seriously taken into account (Frith and de Vignemont, 2005). For instance, Nadelhoffer and Feltz (2008) showed that responses during moral dilemma evaluation differed when evaluators were agents in the question rather than observers. Along the same line, distinct brain regions are activated during participants’ intuition about their own and others’ moral transgression (Berthoz et al., 2006). Such difference may explain the variation in the degree of utilitarianism of responses to various dilemmas inducing an abstract judgment (reaction to moral violation by another person) or a choice of action (i.e., from a first person perspective; Monin et al., 2007). It could also explain why people acknowledge moral norms and make appropriate moral judgment but fail to act accordingly, illustrating a capacity for “moral hypocrisy” (Batson et al., 1997; Valdesolo and DeSteno, 2007). Data from studies of psychopathic individuals and patients with brain lesions who bear intact moral judgment yet consistently commit immoral acts further suggest this possible dissociation (Eslinger and Damasio, 1985; Glenn et al., 2009a,b; Tassy et al., 2009; Cima et al., 2010). However, to our knowledge, a clear experimental evidence of distinct responses to dilemmas with questions targeting both hypothetical judgment and choice of action is still lacking^1^.

The present study explores empirically and systematically if decisions made in the context of moral judgment or of moral choice of action differ, and how variations in the contextual framing of the dilemma participate to this difference. We hypothesize that if judgment and choice of action are influenced differently by variations of qualitative parameters framing the moral dilemma, it might suggest that they rely on distinct cognitive processes, as recently proposed in a neuroscientific experiment (Tassy et al., 2012). Because these two parameters have been shown to influence decision during moral dilemma evaluation, in the present experiment we varied: (i) the *number of people benefiting* from the potential decision (utilitarian preference), with the hypothesis that one favors unknown individuals when they are a substantial number therefore maximizing aggregate welfare (Petrinovich et al., 1993; Shenhav and Greene, 2010; Bartels and Pizarro, 2011) and therefore overriding our tribal instinct (Boyd et al., 2011), and reflecting the development of rules at the heart of complex societies (Hayek, 1979; Tassy et al., 2007); and (ii) the degree to which the potential victim of the scenario is related to the participant/decision maker (*closeness of relationship*) with the hypothesis that we favor the individual(s) that are socially close to us because of cognitive mechanisms at stake when human beings evolve in restricted groups (Richerson and Boyd, 2001; Thomas et al., 2011; Kurzban et al., 2012).

## Materials and Methods

### Participants

Two hundred and forty students volunteered in the experiment and were randomly divided into 8 groups of 30. Groups were matched on the basis of age, gender, education level, mother tongue, number of siblings, and religious practice.

### Materials

Stimuli consisted of 15 dilemmas with moral content and 9 non-moral control dilemmas. The basic framework of dilemmas scenarios was either directly inspired from a previous battery developed by Greene et al. (2001), adapted to take into account cultural differences and validated by the experimenters in a previous study (Tassy et al., 2012). The nine non-moral dilemmas required decision making in situations with no moral connotation whatsoever (cf. Appendix).

Eight different versions of each moral dilemma (A, B, C, D, E, F, G, H), for a total of 120 moral dilemmas, were presented in eight different questionnaires. Each group of participants therefore completed one of the eight questionnaires.

Versions A, B, C, and D: the independent variable was the number of people that could potentially benefit from the judgment and choice of action, ranging from less than four (versions A) to hundreds or thousands (Versions D). The figures were adapted to ensure plausibility of the scenarios. In all versions the potential victim of the decision had no affective proximity with the evaluator.
Versions E, F, G, and H: the independent variable was the affective proximity between the participant who had to make a decision and the potential victim(s) of the decision. Versions E always involved a first-degree relative (father, brother, sister, son, mother, daughter, …); versions F a second degree relative (cousin, uncle, …); versions G a close friend or relation; versions H involved unknown individuals with no affective or genetic proximity (e.g., the baker). The number of people that would potentially benefit from the decision was kept constant throughout the versions.

Non-moral dilemmas were identical in all questionnaires. The order of presentation of moral and non-moral dilemmas was counterbalanced within and between questionnaires.

### Task and data collection

Anonymous paper and pencil questionnaires were used. The questionnaire started with socio-demographic questions to collect anonymous data on gender, familyhood, age, level of education, work, marital status, children, religion, and mother tongue.

After reading each dilemma, the participant had to answer successively two questions by Yes or No: “*Is it acceptable to* …*?*” (Judgment condition) and “*Would you* …*?*” (Choice of action condition).

Participants were instructed to imagine each hypothetical situation as vividly and realistically as they could and to be as honest as possible in their decision. In addition, we insisted on the anonymous nature of the questionnaires and instructed participants not to go back to check and/or change any of their prior responses.

### Ethics statement

Written informed consent was obtained from all participants. The French Law does not require approval of an ethics committee when data from a questionnaire are collected and analyzed anonymously.

### Data processing

For non-moral dilemmas, “appropriate” responses implied the maximization of beneficence overall consequences (e.g., buying a new television instead of repairing the old one for the same price was coded 1 and “inappropriate” was coded 0). For moral dilemmas, response to each question was coded 1 if it favored maximizing the good of more people at the expense of very few identified individuals (“utilitarian” response; e.g., sacrificing one person’s life to save five), and zero for the reverse situation.

For the analysis of the difference of responses between conditions (Judgment-Choice of action) for each dilemma, we quoted 1 when judgment response was utilitarian but choice was not, −1 for the reverse situation and 0 when judgment and choice responses were identical.

Statistical analyses were performed using SPSS software Version 17.0 (SPSS Inc., Chicago, IL, USA).

Probabilities of utilitarian responses in judgment and choice of action conditions were calculated for all dilemmas.

We compared responses between judgment and choice conditions using paired *t*-tests.

To characterize the effect of the variation of the two variables (i.e., number of lives saved and affective proximity) on both the judgment and choice of action, we performed a logistic regression analysis.

To statistically test the possibility that the two variables could have a different effect on the two conditions, we further performed an ordinal regression on the differences of responses between conditions for each dilemma.

To account for the non-independence of within-subject responses, regression models were fitted using the generalized estimating equations method (Koenigs et al., 2007).

## Results

### Non-moral dilemmas

**Figure 1:**
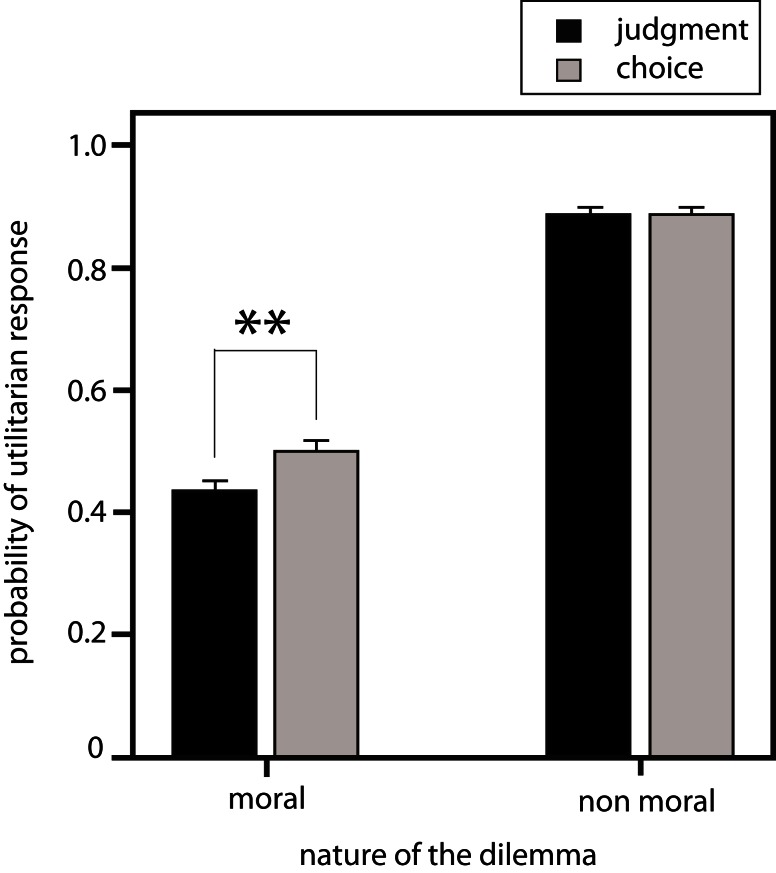
**Overall probability of utilitarian response in both conditions (Gray bars: choice of action condition; black bars: judgment condition)**.

Probabilities of appropriate response were identical in the judgment (appropriate *M* = 0.89; SD = 0.12), and choice (Appropriate *M* = 0.89; SD = 0.11) conditions [*p*(paired *t*-test) = 0.879; Figure 1].

### Moral dilemmas

#### Overall difference between judgment and choice of action

We first determined whether the two dimensions incorporated into the dilemmas (affective proximity and number of people saved) resulted in systematic differences in responses in both conditions (judgment vs. choice of action). The probability of utilitarian response is significantly lower in the judgment condition than in the choice of action condition, whatever the type of variable (judgment condition *M* = 0.43; SD = 0.18; choice condition *M* = 0.51; SD = 0.17; *p* < 0.001; Figure 1). This suggests that participants choose to endorse actions they judge unacceptable.

#### Effect of the variation of the number of people benefiting from the decision

**Figure 2:**
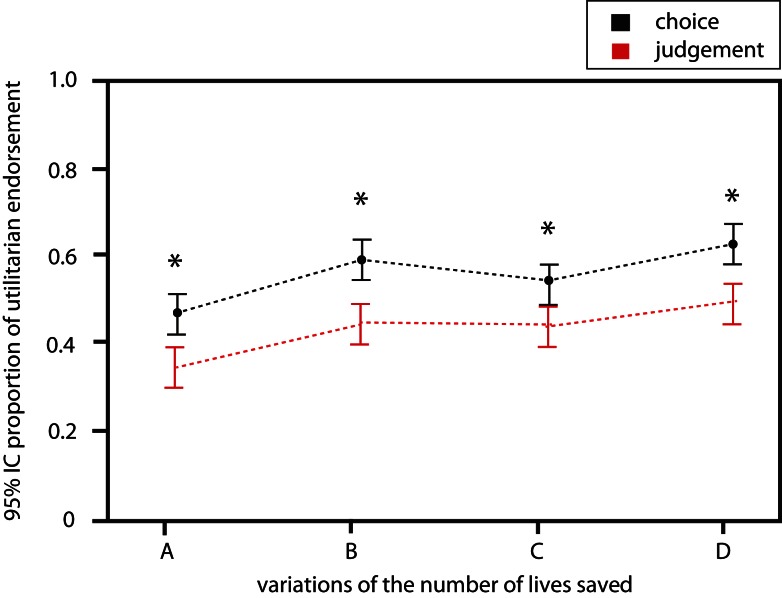
**Influence of the variation of number of live saved on the probability of utilitarian response in both conditions**.

A significant effect of the variations of the number of people benefiting from the decision was observed in both judgment [OR = 1.19 (1.09; 1.29); *p* < 0.001] and choice of action conditions [OR = 1.19 (1.09; 1.30); *p* < 0.001] (Figure 2; Table 1). The more the number of lives saved is high, the more participants tend to be utilitarian in their judgment and choice of action.

**Table 1:** **Probability of utilitarian judgment when varying the number of people benefiting from the decision**.

Variations	Probability of utilitarian judgment (SD)	Probability of utilitarian choice of action (SD)	*p*(Paired *t*-test)
E	0.38 (0.18)	0.30 (0.21)	0.009
F	0.42 (0.19)	0.40 (0.22)	0.464
G	0.43 (0.17)	0.49 (0.18)	0.143
H	0.50 (0.17)	0.58 (0.18)	0.010

As shown by OR values, variations in the number of lives saved seem to influence identically the responses in both conditions. The ordinal regression on the differences of responses between conditions for each dilemma did not yield any significant results [cumulative proportional OR = 0.99, 95% confidence interval (0.91; 1.08); *p* = 0.850]. It further indicates that the effect of people benefiting from the decision is identical in both conditions.

#### Effect of affective proximity variations

**Figure 3:**
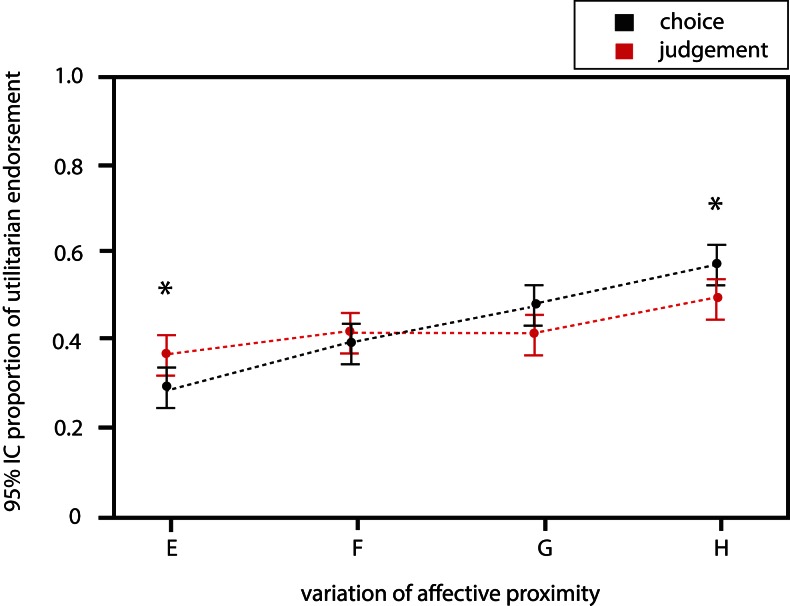
**Influence of affective proximity variations on the probability of utilitarian response in both conditions**.

A significant effect of the variation of affective proximity is observed in both judgment [OR = 1.17 (1.07; 1.27); *p* < 0.001] and choice of action [OR = 1.49 (1.36; 1.62); *p* < 0.001] conditions (Figure 3; Table 2). Moreover, the effect is significantly stronger in the choice of action than in the judgment condition (ORchoice > ORjudgment). This is confirmed by the ordinal regression which revealed a significant influence of affective proximity on the difference of responses between judgment and choice of action [cumulative proportional odds ratio (OR) = 0.80, 95% confidence interval (0.73; 0.87); *p* < 0.001], proving that the effect is indeed different in both conditions. Implication of a close relative has thus a stronger influence on behavioral choice of action.

**Table 2:** **Probability of utilitarian judgment when varying affective proximity**.

Variations	Probability of utilitarian judgment (SD)	Probability of utilitarian choice of action (SD)	*p*(Paired *t*-test)
A	0.35 (0.21)	0.47 (0.2)	0.025
B	0.45 (0.21)	0.60 (0.17)	0.002
C	0.45 (0.21)	0.55 (0.22)	0.006
D	0.50 (0.20)	0.63 (0.20)	0.004

## Discussion

The very high number of appropriates responses for both judgment and choice when facing non-moral dilemmas illustrates that participants were able to make appropriate decisions. Furthermore, in this non-moral context responses were coherent across conditions of judgment and choice of action.

In the case of moral dilemmas, we first observe a dissociation between judgment and choice of action, with responses to choice of action being overall more utilitarian than responses to judgment. Quite surprisingly, participants would thus generally accept to perform an action they have judged as morally unacceptable. Everyone has experienced the potential discrepancy between what one judges as right, from a moral point of view, and what one actually does when a choice between alternative behaviors is to be made. This is coherent with a recent study reporting that a substantial fraction of participants chose an action they judged morally wrong during moral dilemma evaluation (Kurzban’s et al., 2012). Such a discrepancy was also already noted in the field of developmental psychology, with results showing that although a relationship does exist between moral thought and action, correlation between the two was weak and inconsistent (Blasi, 1980). This was recently illustrated experimentally in real dilemmas situations in which participants agreed to inflict more pain to someone than what they judged as being acceptable (FeldmanHall et al., 2012). Moral judgment can thus conflict with effective behavior, supporting the proposition to integrate a separate measure of moral norm in the Theory of Reasoned Action (TRA) to enhance its predictive utility (Manstead, 2000). This discrepancy between a judgment and the associated act also resonates with what is termed Akrasia in the philosophical literature (i.e., acting against one’s own judgment). To explain such phenomena, Davidson (2001) proposed that the mind could be partitioned into two quasi-independent systems: one that finds a certain course of action to be best all things considered, and another that prompts a different course of action. Our results suggest that this seems to be especially true in the context of decision making during hypothetical moral dilemma evaluation and that the process at play during moral evaluative judgment and subjective action choice could be at least partially different. At the cerebral level, previous results of a rTMS study using identical dilemmas and tasks suggest that right dorsolateral prefrontal cortex (rDLPFc) disruption alters moral judgment but not choice (Tassy et al., 2012). Moral judgment thus involves a brain network in which the rDLPFc is required to process allocentric integration of contextual information. By contrast, the fact that psychopathy characterized by serious immoral behavior and a dysfunction of the ventromedial prefrontal cortex leaves moral judgment intact (Cima et al., 2010) suggest that moral action choice would mainly rely mainly on VMPFc function.

Our second observation is that the more the number of lives saved is high, the more participants tend to be utilitarian in their judgment and choice of action. This result is in line with findings from previous studies on moral judgment studying responses to a single question close to our choice of action condition (O’Neill and Petrinovich, 1998; Shenhav and Greene, 2010). It suggests that cognitive processes (either common or distinct) that lead to judgment or choice of action are identically influenced when participants engage in strict cost-benefit analysis (“expected moral value”) i.e., balancing the cost for the potential victim against the benefit for an increasing number of people of the decision (Shenhav and Greene, 2010). However, as illustrated in Figure 1, the probability of utilitarian responses is constantly higher for the choice of action than for judgment. This may seem counterintuitive at first glance, as choice of action should imply more personal consequences and thus the sacrifice of someone should be more affectively laden. A possible explanation could be that the mechanism involved in choice of action overlap considerably with those engaged in self-interested decisions (Shenhav and Greene, 2010). Because it is rewarding to save a maximum number of people choice of action would be less influenced by emotions, and thus more utilitarian (“economically rational”). Oppositely, the psychological mechanism involved in judgment is an impersonal evaluation of domain-general contextual information strongly influenced by cultural norms and secondary prosocial emotions integration (Moll et al., 2005; Tassy et al., 2012). These secondary emotions would come into play and bias judgment responses toward less utilitarianism.

As expected from results of previous psychological studies, participants’ decisions are generally less utilitarian when potential victims are more affectively related to them (O’Neill and Petrinovich, 1998). This is true for both judgment and choice. Interestingly however, the effect is significantly stronger in the choice of action than in the judgment condition (ORchoice > ORjudgment), which reveals that affective proximity influences more choice of action than judgment, as also reported by Kurzban et al. (2012). A potential explanation could be that implication of a kin has strong personal consequences that are most decisive in action choice (Thomas et al., 2011). Indeed, action choice entails self-relevant consequences whereas making judgments mostly relies on an impersonal objective evaluation of the situation that has no personal consequences (Sood and Forehand, 2005). The stronger effect of affective proximity on choice has a consequence: while the probability of utilitarian responses is higher for choice of action than for judgment in the case of low affective proximity, an opposite effect is observed for close affective proximity.

This suggests that choice of action becomes less utilitarian than judgment as the level of personal consequences worsen, most likely because of a greater influence of primary emotional contextual information (Greene et al., 2001). Judgment would be less prone to such primary emotions elicited by taking into account personal consequences. This differential influence of a single parameter on judgment and choice of action is coherent with a recent rTMS study suggesting that objective evaluative judgment and subjective action choice during decision making in the context of moral dilemma rely on distinct cognitive processes (Tassy et al., 2012).

## Conclusion

The present study provides empirical evidence that abstract judgment and hypothetical choice of action are differently influenced by variations of contextual parameters during moral dilemma evaluation. This provides an experimental evidence for a divergence between judgment and choice of action during moral evaluation, suggesting that moral judgment and moral choice may be underlied by distinct psychological mechanisms (Tassy et al., 2012). This finding may shed new light on the mechanisms behind some of our most important social decisions, namely policy decisions involving affect and life and death stakes for large numbers of people such as what happened during 9/11 when the US authorities had to decide, at least for the second plane, whether or not they should shoot down a commercial plane full of civilians in order to save thousands of individuals stuck in the Twin Towers. This differentiation between evaluative judgments and actual choices of action opens a way for exploring moral cognition, notably to allow better understanding of pathologies that affect social skills. In this respect, such a battery of moral dilemmas and their variations in two dimensions may be useful for future research aimed at evaluating moral cognition, especially in the case of psychopathic behaviors.

## Conflict of Interest Statement

The authors declare that the research was conducted in the absence of any commercial or financial relationships that could be construed as a potential conflict of interest.

## Footnotes

^1^Our study is not immune to the usual critic that can be raised in this kind of settings involving moral dilemmas: here what we consider an action is obviously what the participants think their action could be if they were to make the decision in real life. For ethical reason, using questionnaire is as far as we can go given the life and death nature of the dilemmas.

## Appendix

### Stimuli

For review purpose, we translated from French to English one moral dilemma and one control dilemma.

*Example of the eight versions of one Moral dilemma* (Each participant respond to only one of the eight versions).

#### Affective proximity variations (A, B, C, D)

##### A

*Votre frère* vient de tomber du bateau de sauvetage en mer dont vous êtes le capitaine. Si vous faites demi-tour pour aller le chercher, vous ne pourrez pas sauver les 4 marins d’un bateau naufragé.

Est-il acceptable d’abandonner *son frère* pour secourir 4 marins?

Abandonnez-vous *votre frère* pour secourir 4 marins?

*English*: *Your brother* fell off a coastguard boat of which you are the captain. If you turn back to rescue him, you will not be able to save four sailors of a sinking boat.

Is it acceptable to abandon *your brother* to save the four sailors?

Do you abandon *your brother* to rescue the four sailors?

##### B

*Votre cousin* vient de tomber du bateau de sauvetage en mer dont vous êtes le capitaine. Si vous faites demi-tour pour aller le chercher, vous ne pourrez pas sauver les four marins d’un bateau naufragé.

Est-il acceptable d’abandonner *votre cousin* pour secourir 4 marins?

Abandonnez-vous *votre cousin* pour secourir 4 marins?

*English*: *Your cousin* fell off a coastguard boat of which you are the captain. If you turn back to rescue him, you will not be able to save four sailors of a sinking boat.

Is it acceptable to abandon *your cousin* to save the four sailors?

Do you abandon *your cousin* to rescue the four sailors?

##### C

*Un de vos copains* vient de tomber du bateau de sauvetage en mer dont vous êtes le capitaine. Si vous faites demi-tour pour aller le chercher, vous ne pourrez pas sauver les 4 marins d’un bateau naufragé.

Est-il acceptable d’abandonner *un de vos copains* pour secourir 4 marins?

Abandonnez-vous *un de vos copains* pour secourir 4 marins?

*English*: *One of your friend* fell off a coastguard boat of which you are the captain. If you turn back to rescue him, you will not be able to save four sailors of a sinking boat.

Is it acceptable to abandon *one of your friends* to save the four sailors?

Do you abandon *one of your friends* to rescue the four sailors?

##### D

*Un marin* vient de tomber du bateau de sauvetage en mer dont vous êtes le capitaine. Si vous faites demi-tour pour aller le chercher, vous ne pourrez pas sauver les 4 marins d’un bateau naufragé.

Est-il acceptable d’abandonner *un marin* pour secourir 4 marins?

Abandonnez-vous *un marin* pour secourir 4 marins?

*English*: *A sailor* fell off a coastguard boat of which you are the captain. If you turn back to rescue him, you will not be able to save four sailors of a sinking boat.

Is it acceptable to abandon *a sailor* to save the four sailors?

Do you abandon *a sailor* to rescue the four sailors?

#### Number of beneficiaries variations (E, F, G, H)

##### E

Un marin vient de tomber du bateau de sauvetage en mer dont vous êtes le capitaine. Si vous faites demi-tour pour aller le chercher, vous ne pourrez pas sauver les *2* *marins* d’un bateau naufragé.

Est-il acceptable d’abandonner un marin pour secourir *2* *marins*?

Abandonnez-vous un marin pour secourir *2* *marins*?

*English*: A sailor fell off a coastguard boat of which you are the captain. If you turn back to rescue him, you will not be able to save *two sailors* of a sinking boat.

Is it acceptable to abandon a sailor to save the *two sailors*?

Do you abandon a sailor to rescue the *two sailors*?

##### F

Un marin vient de tomber du bateau de sauvetage en mer dont vous êtes le capitaine. Si vous faites demi-tour pour aller le chercher, vous ne pourrez pas sauver les *8 marins* d’un bateau naufragé.

Est-il acceptable d’abandonner un marin pour secourir *8* *marins*?

Abandonnez-vous un marin pour secourir *8 marins*?

*English*: A sailor fell off a coastguard boat of which you are the captain. If you turn back to rescue him, you will not be able to save *eight sailors* of a sinking boat.

Is it acceptable to abandon a sailor to save the *eight sailors*?

Do you abandon a sailor to rescue the *eight sailors*?

##### G

Un marin vient de tomber du bateau de sauvetage en mer dont vous êtes le capitaine. Si vous faites demi-tour pour aller le chercher, vous ne pourrez pas sauver les *dizaines de marins* d’un bateau naufragé.

Est-il acceptable d’abandonner un marin pour secourir des *dizaines de marins*?

Abandonnez-vous un marin pour secourir des *dizaines de marins*?

*English*: A sailor fell off a coastguard boat of which you are the captain. If you turn back to rescue him, you will not be able to save *tens of sailors* of a sinking boat.

Is it acceptable to abandon a sailor to save *tens of sailors*?

Do you abandon a sailor to rescue *tens of sailors*?

##### H

Un marin vient de tomber du bateau de sauvetage en mer dont vous êtes le capitaine. Si vous faites demi-tour pour aller le chercher, vous ne pourrez pas sauver les *centaines de marins* d’un bateau naufragé.

Est-il acceptable d’abandonner un marin pour secourir les *centaines de marins*?

Abandonnez-vous un marin pour secourir les *centaines de marins*?

*English*: A sailor fell off a coastguard boat of which you are the captain. If you turn back to rescue him, you will not be able to save *hundreds of sailors* of a sinking boat.

Is it acceptable to abandon a sailor to save the *hundreds of sailors*?

Do you abandon a sailor to rescue the *hundreds of sailors*?

#### Example of one non-moral dilemma

Vous allez dans une librairie pour acheter 50 € de livres. Vous avez un bon de réduction de 25% qui expire aujourd’hui. Vous avez un bon de réduction de 30% qui expire dans un an, donc vous pourrez l’utiliser plus tard.

Est-il acceptable d’utiliser le bon de 25% de réduction, plutôt que celui de 30% qui expire dans 1 an?

Utilisez-vous le bon de 25% de réduction, plutôt que celui de 30% qui expire dans 1 an?

*English*: You go into a bookstore to buy for 50 € pounds. You have a coupon of 25% which expires today. You also have a coupon of 30% which expires in a year, so you can use it later.

Is it acceptable to use the correct 25% discount coupon, rather than the 30% coupon that expires in 1 year?

Do you use the 25% discount coupon, rather than the 30% coupon that expires in 1 year?
